# *Bifidobacterium longum* CCM 7952 Promotes Epithelial Barrier Function and Prevents Acute DSS-Induced Colitis in Strictly Strain-Specific Manner

**DOI:** 10.1371/journal.pone.0134050

**Published:** 2015-07-28

**Authors:** Dagmar Srutkova, Martin Schwarzer, Tomas Hudcovic, Zuzana Zakostelska, Vladimir Drab, Alena Spanova, Bohuslav Rittich, Hana Kozakova, Irma Schabussova

**Affiliations:** 1 Laboratory of Gnotobiology, Institute of Microbiology of the Czech Academy of Sciences, v.v.i., Novy Hradek, Czech Republic; 2 Laboratory of Cellular and Molecular Immunology, Institute of Microbiology of the Czech Academy of Sciences, v.v.i., Prague, Czech Republic; 3 Dairy Research Institute Ltd., Prague, Czech Republic; 4 Brno University of Technology, Faculty of Chemistry, Brno, Czech Republic; 5 Institute of Specific Prophylaxis and Tropical Medicine, Center for Pathophysiology, Infectiology and Immunology, Medical University of Vienna, Vienna, Austria; INSERM, FRANCE

## Abstract

**Background:**

Reduced microbial diversity has been associated with inflammatory bowel disease (IBD) and probiotic bacteria have been proposed for its prevention and/or treatment. Nevertheless, comparative studies of strains of the same subspecies for specific health benefits are scarce. Here we compared two *Bifidobacterium longum* ssp. *longum *strains for their capacity to prevent experimental colitis.

**Methods:**

Immunomodulatory properties of nine probiotic bifidobacteria were assessed by stimulation of murine splenocytes. The immune responses to *B*. *longum* ssp. *longum* CCM 7952 (Bl 7952) and CCDM 372 (Bl 372) were further characterized by stimulation of bone marrow-derived dendritic cell, HEK293/TLR2 or HEK293/NOD2 cells. A mouse model of dextran sulphate sodium (DSS)-induced colitis was used to compare their beneficial effects *in vivo*.

**Results:**

The nine bifidobacteria exhibited strain-specific abilities to induce cytokine production. Bl 372 induced higher levels of both pro- and anti-inflammatory cytokines in spleen and dendritic cell cultures compared to Bl 7952. Both strains engaged TLR2 and contain ligands for NOD2. In a mouse model of DSS-induced colitis, Bl 7952, but not Bl 372, reduced clinical symptoms and preserved expression of tight junction proteins. Importantly, Bl 7952 improved intestinal barrier function as demonstrated by reduced FITC-dextran levels in serum.

**Conclusions:**

We have shown that Bl 7952, but not Bl 372, protected mice from the development of experimental colitis. Our data suggest that although some immunomodulatory properties might be widespread among the genus *Bifidobacterium*, others may be rare and characteristic only for a specific strain. Therefore, careful selection might be crucial in providing beneficial outcome in clinical trials with probiotics in IBD.

## Introduction

Inflammatory bowel disease (IBD), such as ulcerative colitis (UC) and Crohn’s disease (CD), comprises a variety of chronic immune-mediated inflammatory disorders of the gastrointestinal tract. Although their aetiology and pathogenesis are not completely understood, it is becoming clear that the combination of genetic, immunological, environmental, and microbial factors play an important role in the development and progression of these conditions [1, 2].

Once considered rather rare, IBD has been raising dramatically over the past few decades in the high income countries [3, 4]. It has been suggested that modern style of life, such as modern sanitation systems, modifications in diet, decline in endemic parasitism, smaller family size or overuse of antibiotics changed the structure and function of the intestinal microbiota [5]. Indeed, dysbiosis, such as reduction in mucosa-associated *Bifidobacterium* spp. or *Lactobacillus* spp., along with an increased relative abundance in pathogenic *Escherichia coli* was observed in IBD patients [6–8]. Current pharmaceutical treatment of IBD, which includes anti-inflammatory and immunosuppressive drugs, biological agents and antibiotics, induces or maintain remission, but is not curative [9]. Moreover, long-term use of these drugs can lead to substantial side effects such as allergic reactions or liver problems [10]. Since IBD is clearly multifactorial and results from complex host-microbiota interactions, preventive strategies targeting the aberrant composition of the intestinal microbiota may have the potential to open new possibilities to tackle these diseases.

Probiotic bacteria, most notably the *Bifidobacterium* and *Lactobacillus* genera have been used for the prevention and/or treatment of gastrointestinal inflammatory diseases [11, 12]. Application of multispecies product VSL#3, which is a mixture of *Lactobacillus*, *Bifidobacterium*, and *Streptococcus* strains, has been used successfully to treat UC [11, 13, 14]. Controversially, clinical trials using different strains have not confirmed the beneficial effects [15–18]. It is getting clear that not only the type of disease and the immunological status of the host, but also the selection of probiotic strain and the mode of application are important factors to be taken into consideration. Essentially, the immunomodulatory potential and beneficial effect of probiotic bacteria seems to be strictly strain-specific and cannot be automatically applied to another strain, or even if to another species.

Several animal models have been developed to understand aetiology and pathogenesis of IBD and to evaluate novel prophylactic/therapeutic strategies [19–21]. Colitis induced by dextran sulphate sodium (DSS) is one of the most extensively used experimental models due to its simplicity, reliability and applicability. Acute or chronic disease can be induced by administration of adaptable concentration of DSS in drinking water [19, 22, 23]. Despite certain differences, DSS model resembles crucial clinical and histopathological features of human IBD; such as changes in morphology of inflamed colon, body weight loss, bloody diarrhoea and aberrant regulatory mechanisms in colon followed by cytokine dysregulation [19, 21, 22, 24].

Other important factors which are shared by human IBD and DSS-induced colitis are reduced expression and/or reorganization of tight junction proteins (e.g. zonulin-1 or occludin) in the epithelium, and increased intestinal permeability for luminal bacteria [25]. The breakdown of the gut barrier function have been shown to precede the clinical relapses in UC patients and also the development of intestinal inflammation in DSS-induced colitis [26].

The genus *Bifidobacterium* is considered as a key member of the human gut microbiota which has been shown to exert a range of beneficial effects on the immune system [27–29]. Notably, the representatives of the species *B*. *longum* are one of the dominant bacterial members of the gut microflora of healthy breast-fed infants [30, 31]. Interestingly, a double blinded randomised controlled clinical trial showed that *B*. *longum* reduced clinical appearance of chronic mucosal inflammation in patients with active UC [32]. Moreover, Fujiwara *et al*. described inhibitory effect of *B*. *longum* strain on DSS-induced experimental colitis [33]. Along these lines, we have shown recently that mucosal application of *B*. *longum* prevented the development of experimental allergy in mice [34, 35]. Although these data suggest that *B*. *longum* might be a promising candidate for prevention/treatment of immune-mediated inflammatory diseases, question remains whether different *B*. *longum* strains, belonging to one subspecies, are equal in their health-beneficial effect.

In the present study, nine different probiotic strains of the genus *Bifidobacterium*, which were originally collected from healthy children and adults, were tested for their ability to induce cytokine production by murine splenocytes. Based on the cytokine profiles, two candidates of one subspecies: i) *Bifidobacterium longum* ssp. *longum* CCDM 372 (Bl 372), the strain with high stimulatory capacity and ii) *B*. *longum* ssp. *longum* CCM 7952 (Bl 7952), the strain with low stimulatory capacity, were selected for further comparative *in vitro* and *in vivo* studies. In experimental model of acute ulcerative colitis, administration of Bl 7952, but not Bl 372, prevented the disruption of gut barrier function by enhanced expression of tight junction proteins in epithelial layer which was associated with reduced development of DSS-induced symptoms.

## Materials and Methods

### Bacterial strains and culture conditions

Nine *Bifidobacterium* strains: *B*. *longum* ssp. *longum* CCDM 372 (Bl 372) and CCM 7952 (Bl 7952); *B*. *longum* ssp. *infantis* CCDM 369 (Bi 369); *B*. *animalis* CCDM 218 (Ban 218) and CCDM 366 (Ban 366); *B*. *adolescentis* CCDM 368 (Bad 368), CCDM 370 (Bad 370), CCDM 371 (Bad 371) and CCDM 373 (Bad 373) were isolated from faecal samples of healthy adults and breast-fed infants and kindly provided by Prof. V. Rada (Department of Microbiology, Nutrition and Dietetics, Czech University of Agriculture, Prague, Czech Republic) and Prof. J. Nevoral (Department of Internal Medicine, 1^st^ Faculty of Medicine, Charles University in Prague, Czech Republic). These strains were deposit in Culture Collection of Dairy Microorganisms (Milcom, Prague, Czech Republic) and Czech Collection of Microorganisms (Brno, Czech Republic). The isolates were cultivated in MRS medium (Oxoid, Hampshire, UK) supplemented with 0.05% L-cysteine-hydrochloride (MRSC) at 37°C in anaerobic conditions for 48 to 72 hours.

### Selection and identification of probiotic strains

Nine bifidobacterial isolates were positively selected on the basis of the resistance to bile salt and low pH (data not shown), further identified and analysed for their immunomodulatory properties in *in vitro* assays. Identification of these isolates was performed by the genus-, species- and subspecies-specific PCR and Amplified Ribosomal DNA Restriction Analysis (ARDRA) as previously described [36, 37]. Please see S1 Materials and Methods for details.

### Inactivation of bacterial strains

Nine probiotic *Bifidobacterium* strains were cultivated in MRSC medium at 37°C in anaerobic condition to the end of exponential growth phase. Bacterial cells were inactivated with 1% phosphate-buffered formalin for 3 h at room temperature, washed 3 times with sterile phosphate buffered saline (PBS) and stored at 4°C as previously described [38].

### Stimulation of mouse splenocytes with inactivated *Bifidobacterium* strains

Immunomodulatory potential of *Bifidobacterium* strains was tested *in vitro* on splenocytes derived from naïve BALB/c mice (8 weeks of age; n = 5) in two independent experiments. Spleens were removed aseptically and single cell suspensions were prepared by disruption of the tissues through a cell strainer into culture medium (RPMI 1640 medium supplemented with 10% heat-inactivated FCS, 10mM HEPES, 100 U/ml penicillin and 100 μg/ml streptomycin). Spleen cells (6 x 10^5^/well) were stimulated with formalin-inactivated *Bifidobacterium* strains (6 x 10^6^/well), Pam3CSK4 (1 μg/ml, InvivoGen, USA) or media alone in 96-well plates at 37°C and 5% CO_2_ for 48 h. Concentration of IFN-γ, TNF-α, IL-6 and IL-10 was determined in cell supernatants by the MILLIPLEX MAP Mouse Cytokine/Chemokine Panel (Millipore Corporation, Billerica, MA, USA) according to manufacturer’s instructions and analysed with Bio-Plex System (Bio-Rad Laboratories, USA).

### Preparation and activation of mouse bone marrow-derived dendritic cells

Mouse bone marrow-derived dendritic cells (BM-DC) derived from naïve BALB/c mice (8 weeks of age; n = 3) were prepared as previously described [34, 39]. Briefly, bone marrow precursors isolated from femurs and tibias were seeded at 2 x 10^5^ cells/ml in RPMI 1640 culture medium containing 10% FCS, 150 μg/ml gentamycin, and 20 ng/ml mouse GM-CSF (Sigma-Aldrich, Germany) and incubated for 8 days. BM-DC (10^6^ cells/well) were stimulated with formalin-inactivated *B*. *longum* strains Bl 7952 and Bl 372 (10^7^ CFU/well), Pam3CSK4 (1 μg/ml), ultrapure LPS (1 μg/ml, InvivoGen, USA) or left untreated for 18 h. The levels of IL-10, IL-12p70, IL-6 and TNF-α were analysed in supernatants of stimulated cells by ELISA using Ready-Set-Go! kits (eBioscience, USA) according to manufacturer’s instructions. For cell surface marker analysis, BM-DC were labelled for 30 min at 4°C with anti-mouse FITC-conjugated CD11c, APC-conjugated MHC II and PE-conjugated CD40, CD80 or CD86 monoclonal antibodies (eBioscience, USA). The data were acquired on a BD FACSAria III flow cytometer (BD Biosciences, USA) and analysed with FlowJo software 7.6.2 (TreeStar, USA).

### Stimulation of Human embryonic kidney 293 cells stably transfected with TLRs and NOD2

Human embryonic kidney (HEK) 293 cells stably transfected with plasmid carrying human (h)TLR2/CD14 gene were kindly provided by Prof. M. Yazdanbakhsh (Leiden, Netherlands), hTLR4/MD2/CD14 were a gift of Prof. B. Bohle, PhD (Vienna, Austria) and hNOD2 expressing cells were purchased from InvivoGen (USA). Cells were stimulated for 20 h with Pam3CSK4 (1 μg/ml), LPS (1 μg/ml), muramyl dipeptide (100 ng/ml, InvivoGen) and formalin-inactivated Bl 7952 and Bl 372 at concentrations of 10^6^, 10^7^, or 10^8^ CFU/ml in 96-well plates. Concentrations of IL-8 were analysed in cell supernatants by ELISA (Thermo Scientific, USA) according to the manufacturer’s instructions.

### Animals

All experimental mice (female 8-week-old BALB/c) were kept in IVC cages (Tecniplast, Italy), exposed to 12: 12-h light-dark cycles, and fed with standard pellet diet (ST1, Bergman, Kocanda, Czech Republic) and tap water *ad libitum*. All experiments were approved by the Animal Experimentation Ethics Committee of the Institute of Microbiology of the Academy of Sciences of the Czech Republic and conducted in accordance with the “European Convention for the Protection of Vertebrate Animals used for Experimental and other Scientific Purposes (CETS No.: 123)”.

### Experimental design and induction of colitis

Female 8-week-old BALB/c mice were divided into 4 experimental groups. Two groups were treated with 200 μl of live bacterial suspension containing 2 x 10^8^ CFUs of Bl 7952 (Bl 7952/DSS; n = 10) or Bl 372 (Bl 372/DSS; n = 8) in PBS by intragastric gavage. Controls (PBS/DSS; n = 10) received PBS only. The administration of bacterial suspensions or PBS was repeated daily for 10 days followed by treatment with 2.5% w/v DSS in drinking water in order to induce acute colitis. Age-matched untreated mice (Naïve; n = 5) were used as healthy controls. Dextran sulphate sodium (DSS, molecular weight 40 kDa; ICN Biomedicals, Cleveland, OH, USA) was administered in the drinking water (2.5% w/v) for 7 days. Clinical symptoms of inflammation were evaluated daily, degree of colitis was determined as disease activity index (DAI) according to Cooper *et al*. [40] with minor modifications (S1 Table). Animals were sacrificed by CO_2_ inhalation and cervical dislocation. The colon was aseptically removed, the length was measured and segments of colon descendens (approximately 0.5 cm in length located 1 cm proximal to the anus) were fixed in 4% buffered paraformaldehyde (Sigma Aldrich, Germany) for histological and immunohistochemical analysis.

### Determination of cytokine response of mesenteric lymph node cells

Mesenteric lymph nodes (MLN) were excised aseptically from all experimental mice; cell suspensions in concentration 6 x 10^6^ cells/ml were prepared and cultivated as described previously [34, 39]. Production of IFN-γ, TNF-α and IL-10 was determined in cell supernatants by the MILLIPLEX MAP Mouse Cytokine/Chemokine Panel (Millipore Corporation, Billerica, MA, USA) according to manufacturer’s instruction and analysed with Bio-Plex System (Bio-Rad Laboratories, USA).

### Histopathological evaluation of inflammation in colonic mucosa

Colon descendens from all experimental mice (one segment from each mouse) were fixed in 4% paraformaldehyde and processed to paraffin blocks as previously described [41]. For determination of inflammation in colonic mucosa and mucin production, the tissue specimens were sliced to 5-μm thickness, deparaffined and stained with haematoxylin and eosin (H&E) or Alcian Blue with post-staining by Nuclear Fast Red (All from Vector, Burlingame, CA, USA). The degree of pathophysiology of the tissue was characterized by presence of ulcerations, damage to the surface epithelium, crypt distortion, signs of oedema, infiltration of inflammatory cells into lamina propria or submucosa and reduction of goblet cells and mucin production according to Cooper *et al*. [40]. The histopathological evaluation was performed blindly by two investigators.

### Immunohistochemical determination of zonulin-1 and occludin in colon

For immunohistochemical staining, the 5-μm deparaffined colon sections (3 sections from each mouse) were treated with protease type XIV (1 mg/ml; Sigma-Aldrich, Germany) for 8 min at 37°C. Endogenous peroxidase was blocked with 0.3% hydrogen peroxide in 100% methanol for 15 min. Nonspecific adsorption was eliminated by incubation of the sections in 10% normal goat serum in PBS for 30 min. Samples were incubated overnight with polyclonal rabbit anti-zonulin-1 (2.5 μg/ml) or anti-occludin (2.5 μg/ml) (ZYMED Laboratories Inc., San Francisco, CA, USA) at 4°C. After washing in PBS, section were incubated with goat anti-rabbit IgG conjugated with horseradish peroxidase (1: 200 in PBS) (Jackson, ImmunoLabs.,West Grove, PA, USA) for 1 hour and stain by AEC chromogen solution (Dako, Carpinteria, CA, USA) for 5 min. The counterstain was carried out with haematoxilin and samples were mounted in the Histotec Aqueous Mountant (Serotec, UK) and viewed under an Olympus BX 40 microscope equipped with an Olympus DP 70 digital camera. Photographs were taken on proposal of Camedia Master 2.5 and DP-Soft (Olympus, Germany).

### Western blot analysis of zonulin-1 and occludin

Segment of colon descendens (approximately 0.5 cm in length located 1.5 cm proximal to the anus) from all experimental mice was homogenized on ice in protein extract buffer (Pierce, Rockford, IL, USA) with a protease inhibitor cocktail (Pierce) for 10 min and sonicated. Samples were centrifuged at 10,000 x rpm for 10 min at 4°C and stored at −80°C until use. Protein concentration was measured using the BCA Protein Assay Kit (Pierce). Western blotting was performed as described by Cinova *et al*. [42] using antibodies against occludin (1:1000) (Invitrogen, Carlsbad, CA, USA), zonulin-1 (1:1000) (ZYMED Laboratories Inc., San Francisco, CA, USA), and β-actin (1:5000) (Abcam, Cambridge, CA, USA). After incubation with the respective primary antibodies, secondary staining was performed using horseradish peroxidase-conjugated species-specific antibodies (1:1000) (ZYMED Laboratories). The reaction was developed using the SuperSignal Weat Femto Maximum Sensitivity Substrate (Thermo Scientific, USA) and the signal intensities were measured on the G:BOX (Syngene, UK) and processed with Adobe Photoshop CS5.

### Evaluation of the intestinal permeability *in vivo*

The intestinal permeability was measured by determination of the amount of FITC-dextran in blood after oral administration as described previously [43]. Briefly, female BALB/c mice were intragastrically gavaged by Bl 7952 (Bl 7952/DSS; n = 5) or PBS (PBS/DSS; n = 5) for ten consecutive days prior to intestinal inflammation was induced by drinking of 2.5% DSS in water for 7 days. Naïve mice served as healthy controls (Naïve; n = 5). On the last day of DSS administration, each mouse received 360 mg/kg of the body weight of FITC-dextran (molecular weight 4.0 kDa; Sigma-Aldrich) by intragastric gavage. Blood samples were obtained after 5 hours, centrifuged at 3,000 x rpm for 30 min, and serum was collected. The concentration of FITC-dextran was determined by spectrophotofluorometry (Safire2, Tecan Group Ltd., Mannedorf, Switzerland) with an excitation wavelength of 483 nm and an emission wavelength of 525 nm using serially diluted FITC-dextran as standard.

### Statistical analysis

Data are expressed as mean ± standard error of the mean (SEM). For the *in vitro* assays, data were analysed by One-way analysis of variance (ANOVA) followed by Dunnett’s multiple comparison post-hoc test. For the *in vivo* assays, unpaired Student’s t-test was used. Statistical analysis was performed using GraphPad Prism 5.0 Software (San Diego, CA, USA).

## Results

### Identification of probiotic *Bifidobacterium* isolates to the species, subspecies and strain level

Nine bacterial isolates of the genus *Bifidobacterium* from healthy children and adults have been selected for their probiotic properties based on resistance to low pH and resistance to bile salt (data not shown). These isolates were identified to the species, subspecies and strain level. PCR-based methods (S1 Materials and Methods) let to identification of three species and two subspecies of the genus *Bifidobacterium*: *B*. *longum* ssp. *longum* CCDM 372 (Bl 372) and CCM 7952 (Bl 7952); *B*. *longum* ssp. *infantis* CCDM 369 (Bi 369), *B*. *animalis* CCDM 218 (Ban 218) and CCDM 366 (Ban 366) and *B*. *adolescentis* CCDM 368 (Bad 368), CCDM 370 (Bad 370); CCDM 371 (Bad 371), and CCDM 373 (Bad 373). Discrimination of the *B*. *longum* strains into subspecies *longum* and *infantis* was performed on the basis of Amplified ribosomal DNA restriction analysis by the enzyme *Sau*3AI (S1 Fig) as described previously [36].

### Immunostimulatory properties of probiotic strains from the genus *Bifidobacterium* are strictly strain-specific

The *in vitro* stimulation of spleen cells collected from naïve BALB/c mice with nine *Bifidobacterium* strains revealed distinct and strain-specific pattern of cytokine production (Table 1). Production of IL-10 by probiotic strains has been associated with their protective effect on inflammatory diseases [44]. Various levels of IL-10 were detected in spleen cultures with values ranging between 100–700 pg/ml, depending on the used strain, where Bi 369, Bad 371, and Bl 372 were the most robust inducers of this anti-inflammatory cytokine (Table 1). On the other hand, Ban 218 and Bad 373 induced low levels of IL-10, but high levels of TNF-α, IL-6 and IFN-γ. Strains Bl 372 and Bad 370 induced substantial production of pro-inflammatory cytokines TNF-α, IL-6, and IFN-γ. Strains Bl 7952 and Ban 366 led to only moderate production of TNF-α and IFN-γ, but significantly elevated levels of IL-10. According to this *in vitro* analysis, we selected two strains of one subspecies *B*. *longum* ssp. *longum*: Bl 372 and Bl 7952, which provided contrasting cytokines pattern and used them for further characterization.

**Table 1:** Cytokine production by splenocytes stimulated with inactivated bacteria of different *Bifidobacterium* strains.

Control/ *Bifidobacterium* strain		IL-10	TNF-α	IL-6	IFN-γ
**Medium**		7 ± 1	6 ± 3	13 ± 4	12 ± 11
**PAM3C**		628 ± 57**	166 ± 20	175 ± 84**	790 ± 332
***B*. *longum***	**Bl 7952**	270 ± 25**	138 ± 12	452 ± 35	173 ± 80
***B*. *longum***	**Bl 372**	532 ± 39**	451 ± 37**	638 ± 80**	744 ± 183
***B*. *infantis***	**Bi 369**	542 ± 85**	384 ± 56**	493 ± 117	108 ± 59
***B*. *animalis***	**Ban 218**	187 ± 14**	935 ± 198**	650 ± 62**	1865 ± 422**
***B*. *animalis***	**Ban 366**	268 ± 14**	200 ± 22	229 ± 13	55 ± 24
***B*. *adolescentis***	**Bad 368**	92 ± 4	189 ± 17	334 ± 52	154 ± 91
***B*. *adolescentis***	**Bad 370**	243 ± 16**	357 ± 36**	923 ± 114**	669 ± 151*
***B*. *adolescentis***	**Bad 371**	606 ± 52**	290 ± 8*	675 ± 70**	145 ± 65
***B*. *adolescentis***	**Bad 373**	186 ± 14*	495 ± 60**	987 ± 87**	1819 ± 275**

Splenocytes isolated from naïve mice (n = 5) were stimulated with formalin-inactivated bifidobacteria (6 x 10^7^ CFU/ml) for 48 h. Pam3CSK4 (PAM3C, 1μg/ml) was use as a positive control. Non-stimulated splenocytes (Medium) were evaluated as control of basal cytokine levels. Concentration of cytokines in supernatants was determined by multiplex assay. Data are expressed as mean ± SEM. Results are representatives of two repeat experiments. Significant difference to medium was calculated using One-way ANOVA and Dunnett’s multiple comparison post-hoc test *p < 0.05; **p < 0.01.

### Strains Bl 7952 and Bl 372 have differential ability to activate dendritic cells *in vitro*

**Fig 1:**
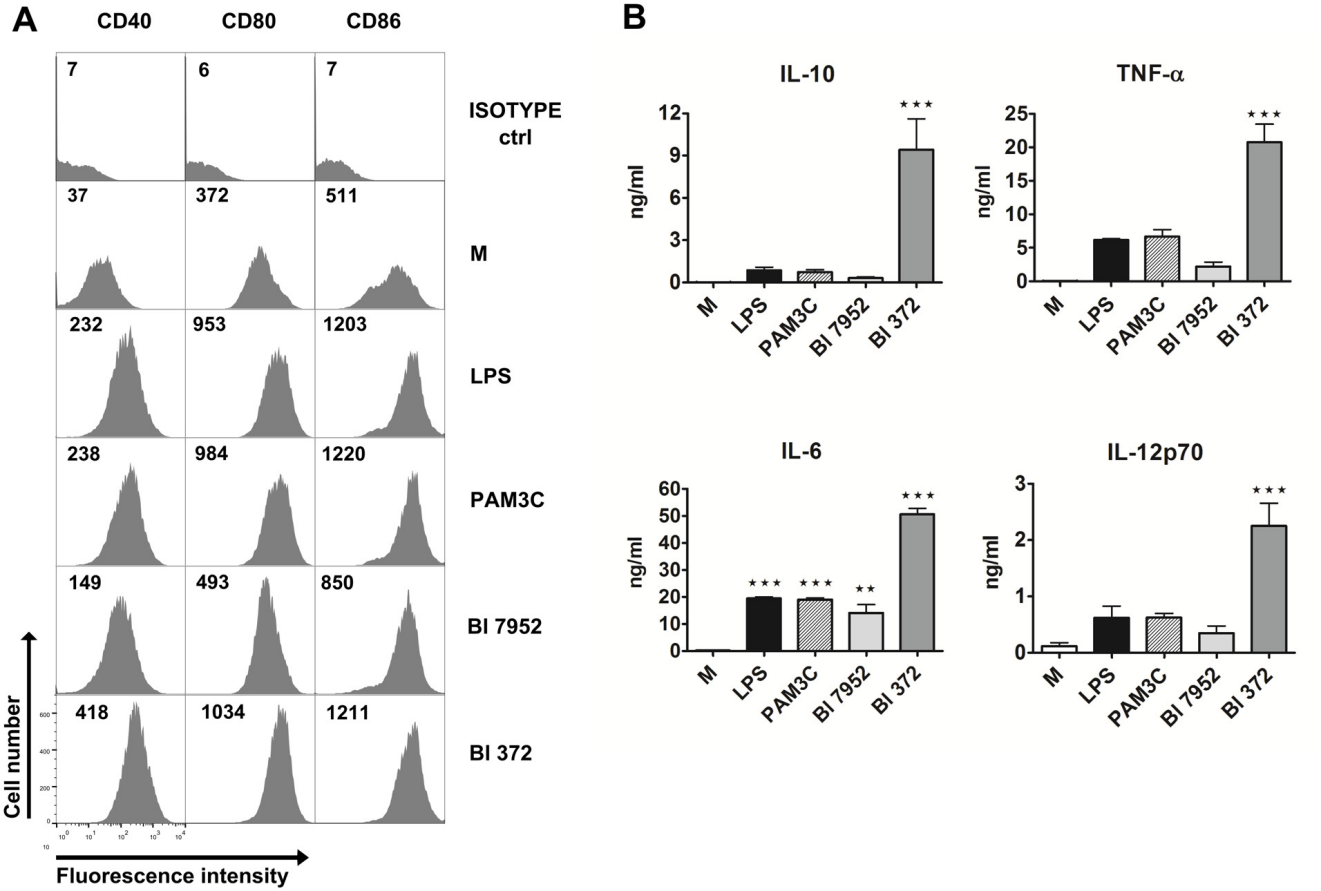
Stimulation of bone marrow-derived dendritic cells with Bl 7952 and Bl 372. Bone marrow-derived dendritic cells (BM-DC) from naïve mice were cultured with formalin-inactivated Bl 7952 or Bl 372 (10^7^ CFU/ml) for 18 h. Ultra-pure lipopolysaccharide from *E*. *coli* (LPS, 1 μg/ml) and Pam3CSK4 (PAM3C, 1 μg/ml) were used as positive controls. Untreated cells (M) served as negative control. (A) Expression of CD40, CD80 and CD86 was assessed by means of flow cytometry. BM-DCs were gated as MHCII+CD11c+. Numbers represent florescence units from one representative experiment out of three. (B) Cytokines in cell culture supernatants were determined by ELISA. Results are representative of three repeat experiments. Data are expressed as mean ± SEM. Significant differences between cytokine levels of experimental group to negative control (M) was calculated using One-way ANOVA and Dunnett’s multiple comparison post-hoc test (**p < 0.01, ***p < 0.001).

Dendritic cells (DC) have been shown to play a central role in regulating intestinal immune homeostasis by induction of tolerance to harmless antigens and commensals or initiating protective immunity against pathogens, contributing to control of intestinal diseases such as inflammatory bowel diseases [45]. In our study, BM-DCs derived from naïve BALB/c mice were used as *in vitro* model to investigate the immunostimulatory potential of both *B*. *longum* spp. *longum* strains. Expression of co-stimulatory markers CD40, CD80, and CD86 were measured to investigate the activation status of BM-DC after stimulation with each bacterial strain. The induction of these surface markers differed between the tested strains. Higher levels of CD40, CD80 and CD86 were observed in DC incubated with Bl 372 than with Bl 7952 (Fig 1A). Levels of IL-10, TNF-α, IL-6, and IL-12p70 were measured in the supernatants of BM-DC stimulated with both *B*. *longum* spp. *longum* strains (Fig 1B). The data show that stimulation of BM-DC with Bl 372 resulted in significantly higher levels of secreted cytokines than stimulation with Bl 7952.

### Both Bl 7952 and Bl 372 signal through TLR2 and NOD2 receptor

**Fig 2:**
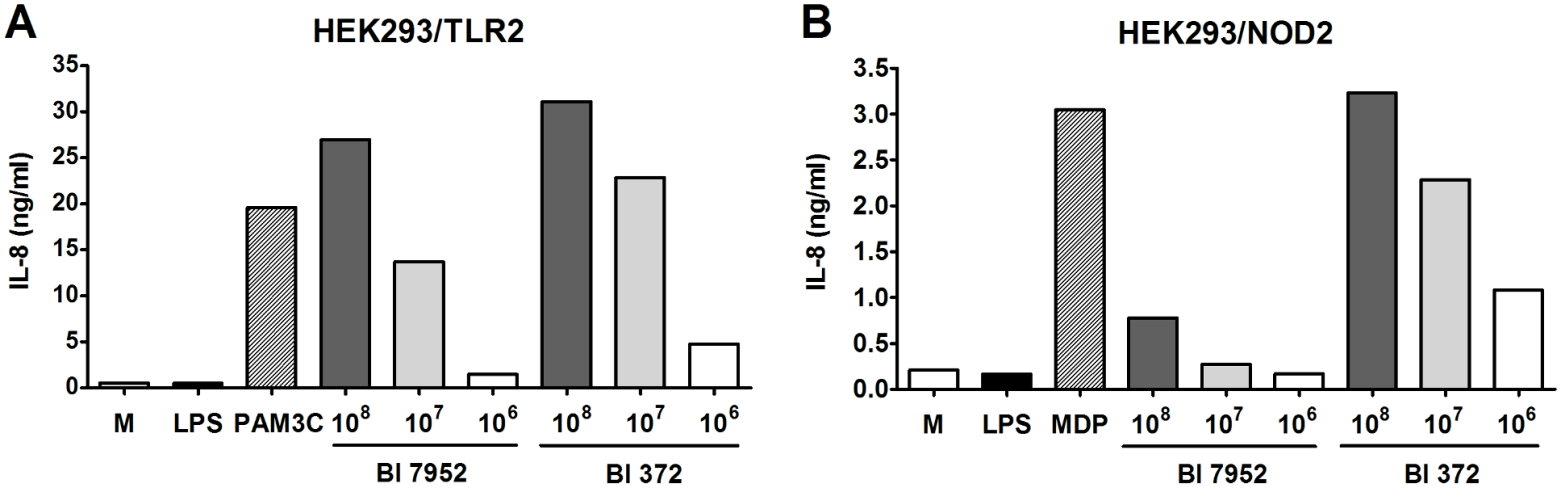
Activation of TLR2 and NOD2 by Bl 7952 and Bl 372. Human embryonic kidney cells (HEK293) stably transfected with an expression vector for human TLR2 (293-hTLR2/CD14) or with NOD2 (293-hNOD2) were stimulated with formalin-inactivated Bl 7952 or Bl 372 for 20 h. Stimulation was performed at concentrations of 10^6^, 10^7^ or 10^8^ CFU/ml. Cells stimulated with ultra-pure lipopolysaccharide from *E*. *coli* (LPS, 1 μg/ml) or untreated cells (M) were used as negative controls. Cells stimulated with Pam3CSK4 (PAM3C, 1 μg/ml) or muramyl dipeptide (MDP, 1 μg/ml) were used as positive controls for TLR2 or NOD2, respectively. Data are expressed as one representative experiment out of three.

To assess the role of TLR2 and NOD2 in recognition of Bl 7952 and Bl 372, HEK293 cells stably transfected with TLR2/CD14 or NOD2 were stimulated with increasing concentrations of both strains. Pam3CSK4 and MDP were used as positive controls for TLR2 and NOD2, respectively. After 20 h of incubation, supernatants were harvested and analysed for IL-8 production. At all three tested concentrations, both Bl 7952 and Bl 372 activated TLR2 in an analogous and dose-dependent manner (Fig 2A). In contrast, stimulation of HEK293/NOD2 with Bl 372 induced markedly higher levels of IL-8 in comparison to stimulation with Bl 7952 (Fig 2B). These results suggest that both Bl 7952 and Bl 372 have similar pattern of usage of TLR2 but distinct patterns of interaction with NOD2. There was no stimulation of HEK293/TLR4 cells with any of *B*. *longum* strains (data not shown).

### Prophylactic application of Bl 7952, but not Bl 372, ameliorates DSS-induced colitis

**Fig 3:**
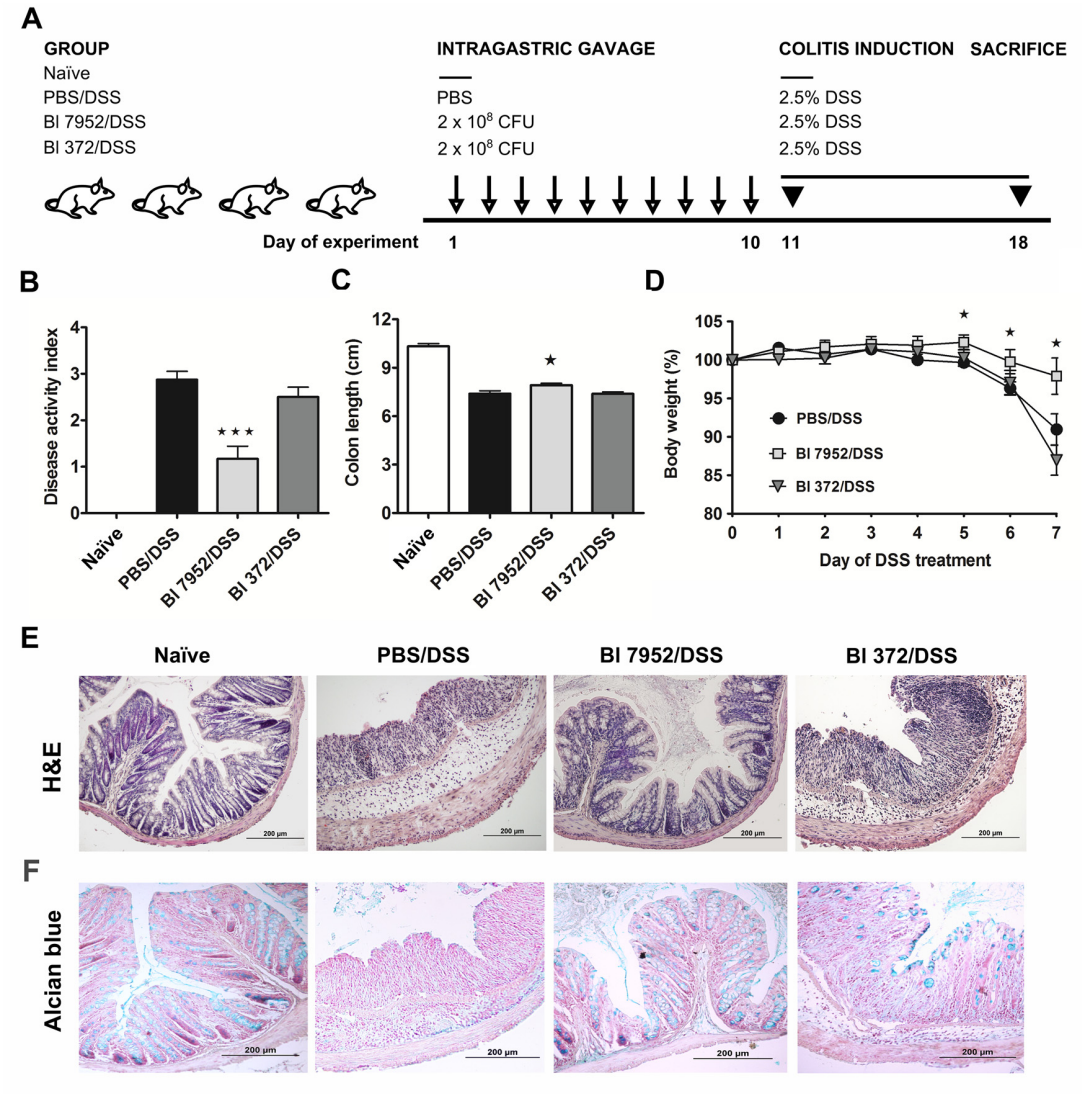
Impact of Bl 7952 and Bl 372 on DSS-induced colitis. (A) Mice were treated with Bl 7952 (n = 10), with Bl 372 (n = 8) or with PBS (n = 10) on ten consecutive days. Naïve animals (n = 5) were left untreated. Colonic inflammation was induced by the addition of 2.5% (w/v) DSS in the drinking water for 7 days. (B) Disease activity index and (C) colon length were evaluated at the end of the experiment. (D) Body weight of mice was evaluated throughout the experiment and the values are expressed as percentage of change of the initial value measured before DSS administration. Changes in colonic mucosa after DSS-treatment are shown on representative histological sections of healthy untreated mice (Naïve), mice treated with PBS (PBS/DSS), Bl 7952 (Bl 7952/DSS) or Bl 372 (Bl 372/DSS). The sections were stained by H&E (E) to address the degree of inflammation and by alcian blue (F) to show the changes in production of mucus in colonic tissue. Graphs show mean ± SEM and represent one out of two experiments. Unpaired Student’s t-test was used for comparison of experimental groups *vs*. control PBS/DSS group (*p < 0.05, ***p < 0.001).

Here we have shown that two strains of one subspecies *B*. *longum* ssp. *longum*, Bl 7952 and Bl 372, possess different immunomodulatory properties *in vitro* (Table 1, Figs 1 and 2). To compare their properties *in vivo*, the mouse model of acute ulcerative colitis induced by administration of 2.5% DSS in drinking water was used. Animals received Bl 7952 or Bl 372 on 10 consecutive days prior to colitis induction (Fig 3A). Disease progression was characterized by weight loss, appearance of diarrhoea or loose faeces and visible faecal blood and summarized in disease activity index (DAI) assessed according to the scale (0–4) of Cooper *et al*. [40] (S1 Table). DSS-treatment increased DAI, reduced the colon length, and reduced body weight in control mice (PBS/DSS) in comparison to naïve animals (Fig 3B–3D). Mice pre-treated with Bl 7952 showed improvement of DAI, and reduction of DSS-induced colon shortening and weight loss compare to PBS/DSS mice (Fig 3B–3D). In contrast, pre-treatment with Bl 372 had no impact on any of these parameters and Bl 372-treated mice did not significantly differ from PBS/DSS group (Fig 3B–3D).

**Fig 4:**
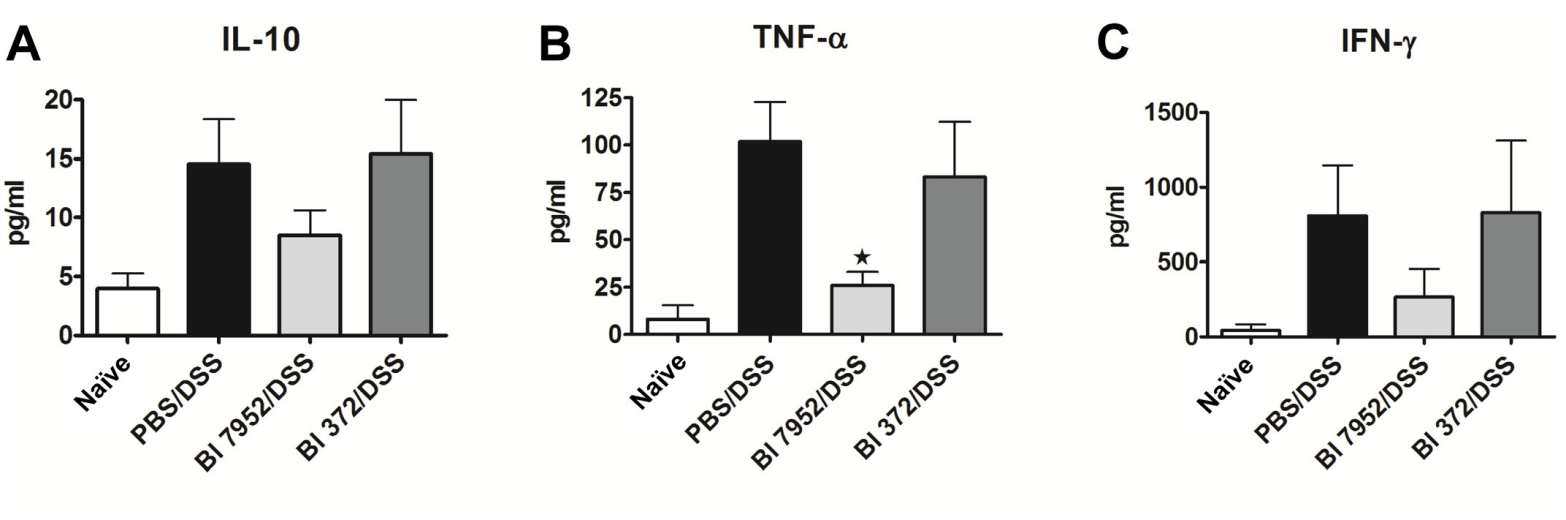
Bl 7952 strain downregulated the secretion of pro-inflammatory cytokines in mesenteric lymph node cells of mice with DSS-induced colitis. Spontaneous production of anti-inflammatory IL-10 (A) or pro-inflammatory cytokines TNF-α (B) and IFN-γ (C) were analysed by multiplex assay in supernatants of mesenteric lymph node cells incubated with media only for 48 h. Data are expressed as mean ± SEM of untreated (n = 5), PBS/DSS (n = 10), Bl 7952/DSS (n = 10) or Bl 372/DSS (n = 8) mice. Unpaired Student’s t-test was used for comparison of experimental groups *vs*. PBS/DSS groups (*p < 0.05).

Histopathological evaluation of the colonic mucosa after DSS-treatment was performed to establish a score (0–4) as described before [40]. The scoring was based on infiltration of inflammatory cells into lamina propria and submucosa, submucosa thickening, and loss of the entire crypt with retained surface epithelium [40, 41]. On sacrifice, histological finding encompassed infiltration of inflammatory cells into lamina propria, thickening of submucosa, loss of epithelial layer and disappearance of mucosal crypt in colonic wall of DSS-treated controls (PBS/DSS; grade 3.5 ± 0.5) and in Bl 372 pre-treated/DSS-treated mice (Bl 372/DSS; grade 3.5 ± 0.3) (Fig 4E). In contrast, Bl 7952-pre-treated mice displayed inhibitory effect on DSS-induced histological changes (Bl 7952/DSS; grade 0.75 ± 0.25) compared to controls. Bl 7952 reduced infiltration of inflammatory cells and pathological changes in mucosa or epithelial layer (Fig 3E).

In control mice with colitis (PBS/DSS) and in mice treated with Bl 372 (Bl 372/DSS), colonic mucin production by goblet cells (Alcian Blue staining) was decreased in comparison to naïve mice (Fig 3F). Markedly, application of Bl 7952 preserved the thinning of the mucus layer and goblet cell depletion (Bl 7952/DSS).

### Administration of Bl 7952 has an impact on the production of cytokines in the mesenteric lymph node cells

Changes in cytokine microenvironment in the gut associated lymphoid tissue, such as mesenteric lymph nodes (MLN), might impact the development of intestinal inflammation in colitis. Therefore, we investigated whether the protective effect of *B*. *longum* Bl 7952 on colitis is associated with changes in production of pro- and anti-inflammatory cytokines. MLN cells collected from naïve, PBS/DSS, Bl 7952/DSS or Bl 372/DSS animals were cultivated for 48 h. Level of cytokines in supernatant was measured by ELISA. We found that pre-treatment with Bl 7952 but not with Bl 372 decreased significantly the production of TNF-α (Fig 4B). Although the levels of IL-10 and IFN-γ were reduced in MLN cell cultures by Bl 7952 in comparison to DSS-controls, the difference did not reach significant level (Fig 4A and 4C).

### Bl 7952 preserves the expression of zonulin-1 and occludin and decreases colon permeability in DSS-treated mice

**Fig 5:**
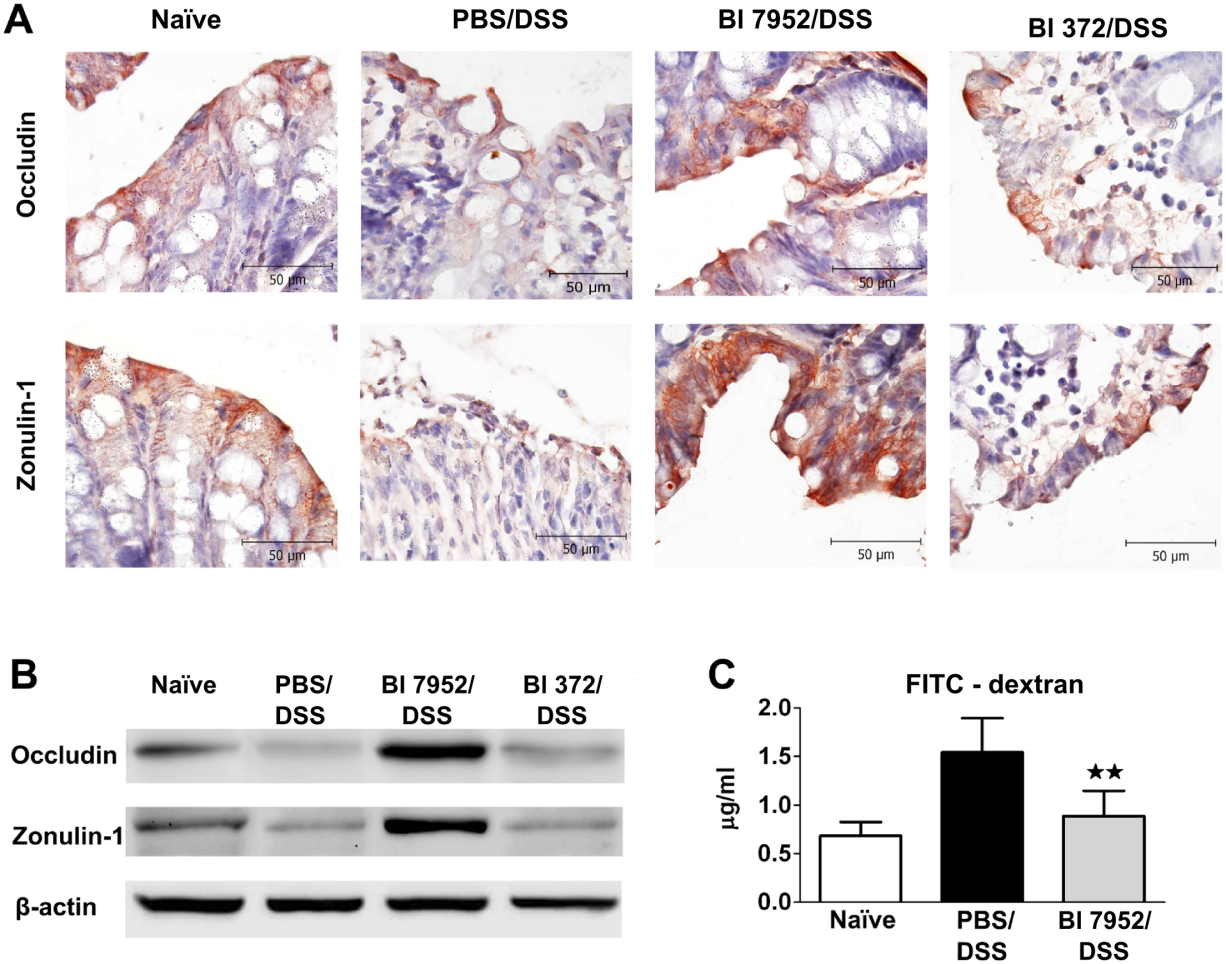
Bl 7952 induces upregulation of zonulin-1 and occludin in colon. Mice were treated with Bl 7952 (n = 10), with Bl 372 (n = 8) or with PBS (n = 10) on ten consecutive days or were left untreated (Naïve; n = 5). Colonic inflammation was induced by addition of 2.5% (w/v) DSS in the drinking water for 7 days. (A) Immunohistochemical detection of occludin and zonulin-1 proteins on representative paraffin-embedded sections of colon. (B) Representative western blotting of occludin and zonulin-1 proteins in the colonic mucosa. Expression of β-actin was used as internal control. (C) Evaluation of intestinal permeability by FITC-dextran. Serum levels of 4.0-kDa FITC-dextran were measured in naïve controls (n = 5), PBS/DSS-treated (n = 5) and Bl 7952/DSS-treated mice (n = 5) 5 hours after its intragastric administration. Data are express as mean ± SEM. Unpaired Student’s t-test was used for comparison of Bl 7952/DSS group *vs*. control PBS/DSS group (**p < 0.01).

Altered intestinal barrier function (breakdown or impairment of the epithelial barrier), which is associated with increased intestinal permeability through decreased expression of tight junction proteins, has been implicated as a critical factor in the development of intestinal inflammation in mouse models of colitis or in human IBD. Occludin and zonulin-1 are proteins involved in the maintenance of the integrity of intact tight junction complexes and barrier function [46]. Here we investigated whether pre-treatment with Bl 7952 interferes with the disruption of these tight junction proteins induced by DSS-treatment. As shown by immunohistochemistry staining (Fig 5A) and Western blotting (Fig 5B), expression of occludin and zonulin-1 was reduced in PBS/DSS mice in comparison to naïve animals. There were no differences between levels of these tight junction proteins in PBS/DSS mice and mice pre-treated with Bl 372 (Fig 5A and 5B). In contrast, application of Bl 7952 preserved the loss of expression and alteration of distribution of both proteins (Fig 5A and 5B).

In order to investigate whether preventive application of Bl 7952 could improve the altered gut barrier function in DSS-colitis, single dose of FITC-dextran was administered by gavage and the intensity of fluorescence was measured in serum 5 h later. The data show that pre-treatment with Bl 7952 markedly decreased FITC-dextran serum concentration in comparison to PBS/DSS mice, reaching similar levels found in naïve mice (Fig 5C). Thus, Bl 7952 preserved the expression of tight junction proteins, which was associated with improved intestinal barrier function in DSS-treated mice.

## Discussion

It is clearly established that the altered composition of the intestinal microbiota plays a role in initiating and maintaining of IBD [47]. Several studies have reported reduction in potentially beneficial *Bifidobacterium* species in IBD patients [6, 48]. Moreover, recent study has shown that *B*. *longum* was one of the dominant species decreased in paediatric patients with new-onset Crohn’s disease [49]. Therefore, modulation of the gut habitat with probiotics, particularly species from the genera *Bifidobacterium* and *Lactobacillus*, represents a novel and exciting strategy in prevention/treatment of microbial dysbiosis associated with mucosal inflammation [50].

In this study we investigated immunomodulatory properties of nine *Bifidobacterium* strains which were obtained from faeces of healthy breast-fed infants and healthy adults, and which possess probiotic properties, such as resistance to gastric acidity (low pH) and bile toxicity, conditions simulating those of the gut environment (data not shown). These probiotic strains were classified by PCR based methods on the species, subspecies, and strain level as *B*. *longum* ssp. *longum* and *B*. *longum* ssp. *infantis*, *B*. *adolescentis* and *B*. *animalis*. In order to call a bacterial strain “probiotic”, it should be well-characterized, classified and specified for its effect on human health. Probiotic bacteria exert their beneficial effects in different ways, among which the immunomodulation of local and systemic immune responses is an important mechanism [51]. In this respect, we tested all strains for the ability to induce cytokine production in spleen cell cultures derived from naïve mice. Results indicate that all strains possess intrinsic immunostimulatory potential, but their ability to induce cytokine expression varies significantly from one bacterial strain to other. We have shown that some *Bifidobacterium* strains, such as Bad 368, Ban 366 or Bl 7952, are poor inducers of both pro- and anti-inflammatory cytokines, while other strains belonging to the same species or subspecies, such as Bad 370, Ban 218 or Bl 372, can stimulate high levels of all evaluated cytokines. These strain-specific effects are in accordance with previous observations on human immunocompetent cells [52–55]. Nonetheless, comparative studies on the immunomodulatory properties of *Bifidobacterium* strains of the same species or subspecies are limited [54–56].

Regulatory cytokine IL-10, which can be produced by multiple cell types, has been shown to play an important role in the maintenance of intestinal homeostasis [44]. Mice with defects in IL-10 production spontaneously develop severe intestinal inflammation in conventional conditions [57]. Relevant to this point, it has been reported that intragastric administration of IL-10-producing recombinant *Lactococcus lactis* reduced colitis in DSS-treated or IL-10-/- mice [58]. Therefore, the capacity of probiotic strain to induce production of IL-10 may be one factor contributing to their beneficial effects [44]. We observed that the majority of tested *Bifidobacterium* strains, except for Bad 368, induced significant levels of IL-10 from naïve spleen cell cultures. Along these lines, bifidobacteria have been shown to induced IL-10 also in human monocyte-derived DC [53], PBMC [59, 60] or in human colonic lamina propria DC [52].

Innate immune cells, such as DC, play an important role in orchestrating the appropriate responses to the enteric luminal microbiota [61]. Defects in how DC recognize and respond to gut bacteria may contribute to IBD pathogenesis [62]. Here we compared the effects of Bl 7952 and Bl 372 on the maturation pattern of BM-DC, as well as their ability to induce cytokine secretion. We found that the activation potential of the two strains varied significantly, suggesting their different functional roles. These results are well in line with previous studies which show that stimulation of DC with different strains of *B*. *longum* led to strain-specific production of pro-inflammatory or regulatory cytokines [55].

Although there have been several studies linking immunomodulatory properties of probiotic strain *in vitro* and its ability to prevent experimental colitis in mice, no clear association has been established so far. In an experimental model of TNBS-induced colitis, Foligne *at al*. demonstrated that probiotic strains with high IL-10/IL-12 ratio *in vitro* provided the best protection *in vivo* [63]. Similarly, Kwon *et al*. demonstrated that administration of probiotic mixture with potent anti-inflammatory properties (high levels of the IL-10/IL-12 production ratio) suppressed the progression of experimental colitis in mice [64]. Along these lines, we have shown recently that *B*. *longum* NCC 3001, a probiotic strain with high IL-10/IFN-γ ratio, offered long term protection in a mouse model of birch pollen allergy [35]. Moreover, we have demonstrated that neonatal colonization of germ-free mice with *B*. *longum* ssp. *longum* CCM 7952 prevented experimental sensitization in a mouse model of allergy [34].

In this study we employed both *in vitro* culture system and *in vivo* mouse model of DSS-induced colitis to compare the immunomodulatory potential of two probiotic strains of the genus *Bifidobacterium* which belong to the same subspecies *B*. *longum* ssp. *longum*. We found that the activity of these two strains, Bl 372 and Bl 7952, differs significantly. While Bl 7952, the strain with low stimulatory potential *in vitro* (as demonstrated on stimulated splenocytes and BM-DC), was able to prevent clinical symptoms in a mouse model of DSS-induced colitis, preserved the tight junction proteins expression and protected epithelial barrier function, the strain Bl 372, which induced high levels of cytokines *in vitro*, had no beneficial effect. Our results are consistent with those reported by Mileti *et al*. who tested the effect of application of three different probiotic strains before exposure to DSS, and observed that only one strain, which was characterized by low levels of induced cytokines, reduced severity of DSS-induced colitis [65].

It has recently been shown, that certain probiotic strains exert their immunomodulatory effects through the interaction with TLRs. Administration of *L*. *plantarum* to healthy subjects increased levels of tight junction-associated zonulin-1 and occludin in the duodenal epithelium and this beneficial effect was shown to be dependent on TLR2-signalling [66]. In our study, both Bl 7952 and Bl 372 have been shown to signal through the TLR2. The fact that only Bl 7952 but not Bl 372 was protective, suggests that TLR2 is not the key player in preserving the gut epithelial barrier in our model.

There is a strong body of evidence suggesting the link between NOD2 and the development of IBD [67, 68]. Within the colonic mucosa, NOD2 can be expresses by various cell populations, such as epithelial cells [69]. In a mouse model, *Nod2* deficiency led to an altered composition of the gut microbiota, predisposing mice to colitis [70]. NOD2 senses many types of peptidoglycan-derived muropeptides, which can vary significantly in their capacity to stimulate NOD2 [71]. Fernandez *et al*. showed that anti-inflammatory capacity of probiotic-derived peptidoglycan was linked to the presence of a NOD2 ligand [72]. In our present study, we demonstrate that although both Bl 7952 and Bl 372 possess ligands which are recognised by NOD2, they potential to stimulate NOD2 differ. Still, the role of NOD2 in recognition of these strains *in vivo* remains to be evaluated.

In humans, IBD is associated with increased intestinal permeability and reduced expression of tight junction proteins, [73] which leads to exposure of luminal antigens to the lamina propria [74, 75]. Therefore, several recently proposed therapeutic approaches to treat IBD are focused on enhancing/restoring of gut barrier integrity [76, 77]. It is of interest, that perfusion of lactic acid bacteria into the small intestine of a healthy subject increased the localization of the scaffold zonula occludens protein zonulin-1 and the transmembrane protein occludin [66]. In an experimental model of colitis, probiotic bacteria have been shown to decrease the intestinal permeability and restore gut barrier integrity by modulation of tight junction proteins [43, 78–80]. In this study we showed that Bl 7952 but not Bl 372 increased expression of zonulin-1 and occludin in the intestinal epithelium which was associated with reduced leakiness of colonic epithelium. Thus, our data show that the choice of *Bifidobacterium* strain for specific benefit, such as maintenance of healthy and functional gut barrier should be considered on a strain-by-strain basis, and interspecies extrapolations are not valid.

In conclusion, our data show strictly strain-specific immune effects of *B*. *longum* subspecies. Thus, it is getting clear, that the beneficial effects of one probiotic strain cannot be extended to other bacteria of the same genus, species or even subspecies. Our work shows that prophylactic administration of probiotic strain *B*. *longum* ssp. *longum* CCM 7952 is capable to preserve the disruption of tight junctions proteins associated with ulcerative colitis pathophysiology. Therefore, this bacterial strain plays the role as regulator of the integrity of the intestinal barrier, which might have important implications for understanding of probiotic mechanisms and for the control of intestinal homeostasis.

## Supporting Information

S1 Fig Amplified ribosomal DNA restriction analysis profile. Amplified ribosomal DNA restriction analysis profile of nine studied *Bifidobacterium*strains (*) and six type/collection control strains of corresponding species and subspecies. Dendrogram is generated from restriction of 914 bpamplicon by different enzymes (*Bam*HI, *Nci*I, *Sau*3AI) and based on UPGMA analysis of Pearson correlation coefficients.
(PDF)

S1 Materials and Methods Identification of *Bifidobacterium* strain by PCR-based methods (PDF)

S1 Table Scoring of disease activity index. Scoring of disease activity index is combined score of weight loss, stool consistency and bleeding divided by 3. *Normal stool* = well-formed pellets; *Loose stool* = pasty and semi-formed stool that does not adhere to the anus; *Diarrhoea* = liquid stool that adheres to the anus. Modified according to Cooper *et al*. [1].
(PDF)
