# Supplementary material for: Bifidobacterium longum CCM 7952 Promotes Epithelial Barrier Function and Prevents Acute DSS-Induced Colitis in Strictly Strain-Specific Manner
Source: PLoS One. 2015 Jul 28;10(7):e0134050. doi: 10.1371/journal.pone.0134050 (PMC4517903; doi:10.1371/journal.pone.0134050)
Supplement: S1 Fig — Amplified ribosomal DNA restriction analysis profile of nine studied Bifidobacteriumstrains (*) and six type/collection control strains of corresponding species and subspecies. Dendrogram is generated from restriction of 914 bpamplicon by different enzymes (BamHI, NciI, Sau3AI) and based on UPGMA analysis of Pearson correlation coefficients. (PDF) [file pone.0134050.s001.pdf]

S1 Fig.

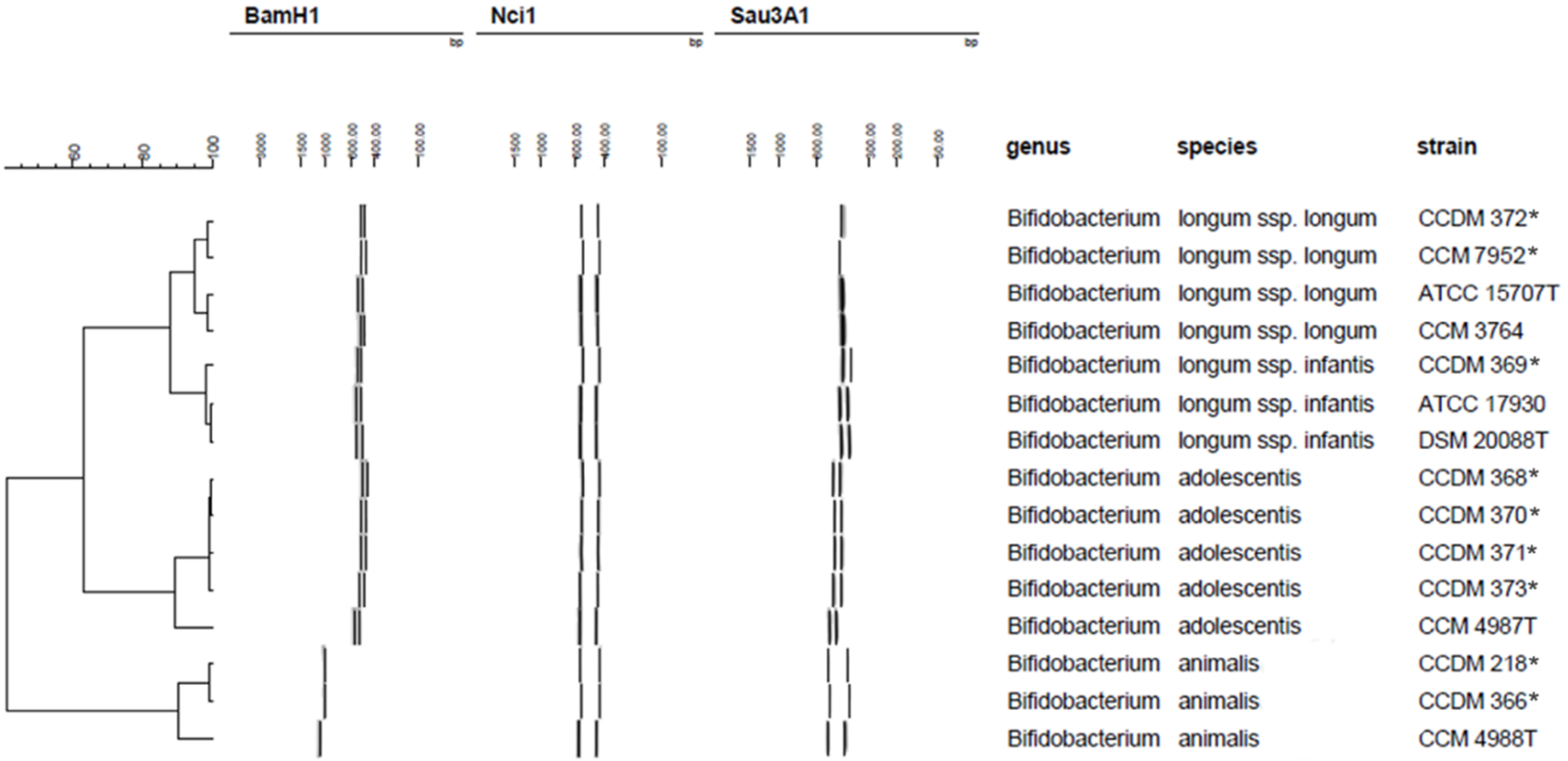

Amplified ribosomal DNA restriction analysis profile. Amplified ribosomal DNA restriction analysis profile of nine studied *Bifidobacterium* strains (*) and six type/collection control strains of corresponding species and subspecies. Dendrogram is generated from restriction of 914 bp amplicon by different enzymes (*Bam*HI, *Nci*I, *Sau*3AI) and based on UPGMA analysis of Pearson correlation coefficients.
