# Supplementary material for: Bifidobacterium longum CCM 7952 Promotes Epithelial Barrier Function and Prevents Acute DSS-Induced Colitis in Strictly Strain-Specific Manner
Source: PLoS One. 2015 Jul 28;10(7):e0134050. doi: 10.1371/journal.pone.0134050 (PMC4517903; doi:10.1371/journal.pone.0134050)
Supplement: S1 Materials and Methods — (PDF) [file pone.0134050.s002.pdf]

S1 Materials and Methods

Identification of *Bifidobacterium* strain by PCR-based methods

Nine *Bifidobacterium* strains which have been selected according to their probiotic properties (survival in conditions of gastric acids and bile salt) were classified by molecular-biological methods as described previously [1]. Briefly, DNA was prepared from crude bacterial cell lysates by phenol (pH 7.8) and chloroform: isoamyl alcohol (24 : 1) extraction according to Sambrook and Russell [2]. The purity and concentration of nucleic acids were confirmed by UV spectrophotometry as described previously [3]. The genus *Bifidobacterium* was confirmed by PCR with specific primers Pbi F1/Pbi R2 described by Roy and Sirois [4]. Ten sets of specific primers were used for identification of bifidobacterial species frequently found in human samples: *B. longum* ssp. *longum*, *B. longum* ssp. *infantis*, *B. dentium*, *B. adolescentis*, *B. bifidum*, *B. breve*, *B. catenulatum/pseudocatenulatum*, *B. angulatum*, *B. gallicum* [5] and *B. animalis* [4]. Amplified ribosomal DNA restriction analysis (ARDRA) of genus-specific PCR product using three endonucelases *Bam*HI, *Nci*I (Takara, Shiga, Japan) and *Sau*3AI (Roche Diagnostic, Manheim, Germany) were used for species and subspecies classification as described previously [1, 3]. Four type and two collection strains used as control in ARDRA analysis were obtained from the Czech Collection of Microorganisms (*B. longum* ssp. *longum* CCM 3764, *B. adolescentis* CCM 4987T, *B. animalis* CCM 4988T), the American Type and Culture Collection (*B. longum* ssp. *longum* ATCC 15707T, *B. longum* ssp. *infantis* ATCC 17930), and the Deutsche Sammlung von Mikroorganismen und Zellkulturen (*B. longum* ssp. *infantis* DSM 20088T). The PCR products were visualised using GelRed™ Nucleic Acid Gel Stain (Biotinum, Hayward, CA, USA) and images were obtained by Fluorescent Image Analyser FLA-7000 (Fujifilm Corporation, Tokyo, Japan). The fingerprinting profile was

analysed by the software programme Gel Compare II (2.0 version) and dendrogram were constructed using UPGMA analysis and Pearson correlation coefficient.

REFERENCES

1. Srutkova D, Spanova A, Spano M, Drab V, Schwarzer M, Kozakova H, et al. Efficiency of PCR-based methods in discriminating Bifidobacterium longum ssp. longum and Bifidobacterium longum ssp. infantis strains of human origin. J Microbiol Methods. 2011;87(1):10-6. doi: 10.1016/j.mimet.2011.06.014. PubMed PMID: 21756944.

2. Sambrook J, Russell DW. Molecular cloning: a laboratory manual. ed. t, editor. Cold Spring Harbor, New York2001.

3. Krizova J, Spanova A, Rittich B. Evaluation of amplified ribosomal DNA restriction analysis (ARDRA) and species-specific PCR for identification of Bifidobacterium species. Syst Appl Microbiol. 2006;29(1):36-44. doi: 10.1016/j.syapm.2005.07.003. PubMed PMID: 16423654.

4. Roy D, Sirois S. Molecular differentiation of Bifidobacterium species with amplified ribosomal DNA restriction analysis and alignment of short regions of the ldh gene. FEMS Microbiol Lett. 2000;191(1):17-24. PubMed PMID: 11004394.

5. Matsuki T, Watanabe K, Tanaka R, Fukuda M, Oyaizu H. Distribution of bifidobacterial species in human intestinal microflora examined with 16S rRNA-gene-targeted species-specific primers. Appl Environ Microbiol. 1999;65(10):4506-12. PubMed PMID: 10508082; PubMed Central PMCID: PMC91600.
