# Supplementary material for: Bifidobacterium longum CCM 7952 Promotes Epithelial Barrier Function and Prevents Acute DSS-Induced Colitis in Strictly Strain-Specific Manner
Source: PLoS One. 2015 Jul 28;10(7):e0134050. doi: 10.1371/journal.pone.0134050 (PMC4517903; doi:10.1371/journal.pone.0134050)
Supplement: S1 Table — Scoring of disease activity index is combined score of weight loss, stool consistency and bleeding divided by 3. Normal stool = well-formed pellets; Loose stool = pasty and semi-formed stool that does not adhere to the anus; Diarrhoea = liquid stool that adheres to the anus. Modified according to Cooper et al. [1]. (PDF) [file pone.0134050.s003.pdf]

S1 Table

Score	Weight loss	Stool consistency	Occult/gross faecal bleeding
**0**	None	Normal	Negative
**1**	1- 5%	Normal	Negative
**2**	5-10%	Loose	Hemoccult positive
**3**	10-20%	Loose	Blood in colon
			Starting bleeding from anus
**4**	>20%	Diarrhoea	Gross bleeding

Scoring of disease activity index. Scoring of disease activity index is combined score of weight loss, stool consistency and bleeding divided by 3. *Normal stool* = well-formed pellets; *Loose stool* = pasty and semi-formed stool that does not adhere to the anus; *Diarrhoea* = liquid stool that adheres to the anus. Modified according to Cooper *et al.* [1].

REFERENCES

1. Cooper HS, Murthy SN, Shah RS, Sedergran DJ. Clinicopathologic study of dextran sulfate sodium experimental murine colitis. Lab Invest. 1993;69(2):238-49. PubMed PMID: 8350599.
